# FVC as an adaptive and accurate method for filtering variants from popular NGS analysis pipelines

**DOI:** 10.1038/s42003-022-03397-7

**Published:** 2022-09-16

**Authors:** Yongyong Ren, Yan Kong, Xiaocheng Zhou, Georgi Z. Genchev, Chao Zhou, Hongyu Zhao, Hui Lu

**Affiliations:** 1 State Key Laboratory of Microbial metabolism, Joint International Research Laboratory of Metabolic & Developmental Sciences, Department of Bioinformatics and Biostatistics, School of Life Sciences and Biotechnology, Shanghai Jiao Tong University, Shanghai, China; 2 SJTU-Yale Joint Center for Biostatistics and Data Science, National Center for Translational Medicine, Shanghai Jiao Tong University, Shanghai, China; 3 Research Affairs, Faculty of Medicine, Chulalongkorn University, Bangkok, Thailand; 4 Department of Biostatistics, Yale University, New Haven, CT USA; 5 Center for Biomedical Informatics, Engineering Research Center for Big Data in Pediatric Precision Medicine, Shanghai Children’s Hospital, Shanghai, China

**Keywords:** Next-generation sequencing, Machine learning, Bioinformatics

## Abstract

The quality control of variants from whole-genome sequencing data is vital in clinical diagnosis and human genetics research. However, current filtering methods (Frequency, Hard-Filter, VQSR, GARFIELD, and VEF) were developed to be utilized on particular variant callers and have certain limitations. Especially, the number of eliminated true variants far exceeds the number of removed false variants using these methods. Here, we present an adaptive method for quality control on genetic variants from different analysis pipelines, and validate it on the variants generated from four popular variant callers (GATK HaplotypeCaller, Mutect2, Varscan2, and DeepVariant). FVC consistently exhibited the best performance. It removed far more false variants than the current state-of-the-art filtering methods and recalled ~51-99% true variants filtered out by the other methods. Once trained, FVC can be conveniently integrated into a user-specific variant calling pipeline.

## Abstract (web summary)

FVC is a method for calling specific gene variants from whole genome data, for potential use in clinical diagnosis and human genetics research.

## Introduction

Whole-genome sequencing (WGS) has been widely used in diagnosing genetic disorders in the pediatrics^1–4^, exploring causative relations with tumor progression^5–7^, studying genetic variation underlying pharmaceutical response^8–10^, performing genome-level comparative analysis^11,12^, assessing gene expression^13–15^, and providing clinical insights and instructions^16–18^. One prominent application with clinical relevance is the utilization of WGS data and bioinformatics tools to identify single nucleotide variants (SNV) and insertion and deletion (INDEL) variants in a single individual genome. The procedure includes at least two main software elements: the variant caller and the variant filter. Variant callers, such as GATK HaplotypeCaller^19,20^, Mutect2^21^, Varscan2^22,23^, and DeepVariant^24^, are utilized to identify the positions and the genotypes of the genomic variants. Variant filters are then applied to eliminate false variants made by the variant caller. This filtering step is necessary due to the fact that there may be tens of thousands of false variants present in the variant call sets.

Current state-of-the-art filtering methods include Frequency^25^, Hard-Filter^20^, VQSR^26^, GARFIELD^27^, VEF^28^, ForestQC^29^ and so on, which employ different strategies in addressing the filtering task. The Frequency model defines variant calls with the variant allelic frequency (VAF) less than 20% or the allelic depth (AD) less than 5 as false variants. The Hard-Filter model applies more user-selected filter conditions to determine the true and false variants^30^. VQSR uses a Gaussian mixture machine learning algorithm to model the technical profile of variants with high quality and low quality and filter out probable false variants^30,31^. GARFIELD uses a deep learning method to learn the different characteristics of true and false variants from a standard cross-validated data set (NA12878^32^). VEF provides a supervised learning method to build a filtering model and predict the probability of the variants to be true. ForestQC filters variants by combining a traditional filtering method and a machine learning approach.

Although these methods address the problem rigorously and are of great utility, some aspects of the available filtering tools and their performance still merit further improvement. For example, the source code of the Frequency method must be modified to adapt to different variant callers. The Hard-Filter, VQSR, and GARFIELD are developed to quality control variant calls identified by GATK. The VEF constructs a filtering model by selecting a subset of features from the existing features in variant calling results. However, in some cases, such as variants identified by Varscan2 and DeepVariant, no feature could be selected from the variant calling results. Thus, these state-of-the-art methods are limited to particular variant callers.

Furthermore, the Frequency method removes all true variants with low variant allelic frequency (VAF < 20%) based on its definition criteria. Hard-Filter removes true variants even when they are very close to the threshold^33^. VQSR is recommended to be used with at least 30 samples^29^, which may not perform well in a single sample. GARFIELD is explicitly designed for whole-exome data^27^. VEF only uses the integer or float format features when constructing the filtering model, but the features in character format are also informative. ForestQC cannot be utilized on single-sample sequencing data. Five of these filtering methods (Frequency, VQSR, Hard-Filter, GARFIELD, and VEF) are available for quality control of variants from single-sample sequencing data and showed high performance in F1-major and accuracy^28^. However, these state-of-the-art methods were unsatisfactory when measured with the Matthews correlation coefficient (MCC) metric^27^, which is a highly suggested measurement for imbalanced data^34^, i.e., the WGS variant calls. Moreover, these five filtering methods had a poor performance in balancing minimizing the filtering of true variants and maximizing the removal of false variants. As a result, the number of eliminated true variants far exceeds the number of removed false variants by using these methods. Therefore, an improved filtering method is required to provide accurate variant call sets derived from a single WGS sample and broaden the scope of the application.

Here, we present an adaptive filtering method FVC (filtering for variant calls) for quality control variant calls from different analysis pipelines. We validated FVC on the genetic variants identified by GATK HaplotypeCaller (abbreviated as GATK), Mutect2, Varscan2, and DeepVariant. Compared to the current state-of-the-art methods, FVC achieved the highest AUC and MCC scores in most cases when assessing with the leave-one-individual-out cross-validation method. We further tested FVC on an additional data set and performed the assessment using the leave-one-chromosome-out cross-validation method. FVC had a consistently superior performance, which has the potential to be used as a general method for quality control variant calls from different analysis pipelines.

## Results

### Construction of FVC

**Fig. 1:**
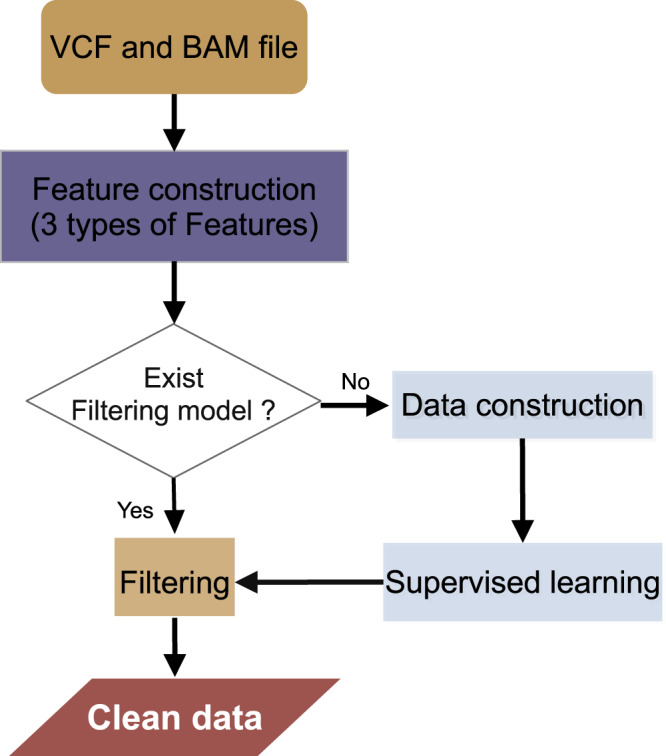
Workflow of FVC. Taking the VCF and BAM files as input, FVC uses the feature construction module to build three types of features related to sequence content, sequencing experiment, and bioinformatic analysis process. If a pre-trained model is already built for the specific pipeline, the variants can be immediately classified as true or false using the filtering module. Otherwise, the pre-trained model can be built using the data construction and supervised learning modules of FVC.

As illustrated in Fig. 1, FVC incorporates four modules: feature construction, data construction, supervised learning, and filtering module. Taking the VCF and BAM files as the input, FVC uses feature construction module to build three types of features related to sequence content, sequencing experiment, and bioinformatic analysis process. If there is no pre-trained model that can be utilized to the user-specific pipeline, an adaptive filtering model is constructed using the data construction module coupled with the supervised learning module. The variant calls are finally labeled as true or false using the filtering module of FVC, and the probability of the variants being true can be found in the INFO field of the output VCF file.

To assess the classification accuracy of the FVC incorporated with different features, different methods of constructing training data, and different machine learning methods, we considered sixteen evaluation metrics and performed the assessments on the gold-standard variant calls derived from WGS datasets (HG001, HG003, HG004, and HG006) at 30× coverage^32,35^. Considering different variant callers may give different initial output, we performed the comparisons on the variant calls identified by GATK, Mutect2, Varscan2, and DeepVariant, separately. All the comparisons were implemented using the leave-one-individual-out cross-validation method. Specifically, sampling was performed four times on the four individuals (HG001, HG003, HG004, and HG006). Each time, a different individual was left out, the genetic variants from the withheld individual formed the test set, and the others formed the training set.

As it can be seen in the Supplementary Tables 1–3 and Supplementary Figs. 1–3, the FVC model containing the constructed features and trained on the imbalanced training data and embedding the XGBoost^36^ as the supervised machine learning method demonstrated the best filtering performance and was incorporated in the final FVC modules (Supplementary Data 1).

### Performance comparison of FVC and the current state-of-the-art methods

After constructing FVC, we performed a head-to-head comparison of FVC with five other state-of-the-art methods (VEF, Frequency, Hard-Filter, VQSR, and GARFIELD) in 4 modes. First, we compared filtering performance with a focus on all SNV or INDEL variants; second, we compared filtering performance with a focus on high-frequency (VAF ≥ 20%) or low-frequency (VAF < 20%) variants; third, we compared filtering performance with a focus on hard-to-detect or easy-to-detect variants; and fourth, we compared filtering performance with a focus on coding or non-coding variants. The comparisons were performed on the variants derived from 4 individuals (HG001, HG003, HG004, HG006) using the leave-one-individual-out cross-validation method. To remove possible bias, we performed the comparisons using the leave-one-chromosome-out cross-validation method and validated the filtering performance on an additional dataset (HG007).

**Fig. 2:**
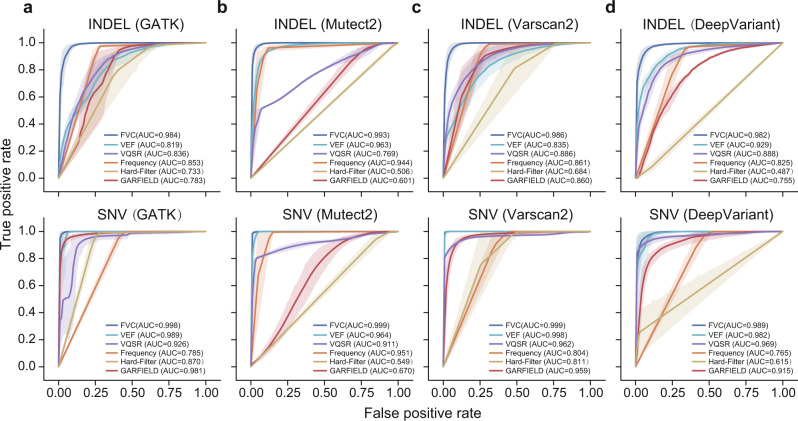
The performance of different filtering methods when applied to SNV or INDEL variants. The SNV and INDEL variants used as testing data are derived from whole-genome sequencing datasets (HG001, HG003, HG004, and HG006) at 30× coverage. The performance of different methods is assessed on the SNV and INDEL variants identified by **a** GATK HaplotypeCaller; **b** Mutect2; **c** Varscan2; and **d** DeepVariant. The performance is assessed by using the leave-one-individual-out cross-validation method. The shaded area indicates the 95% confidence intervals (*n* = 4 biologically independent samples). FVC consistently achieves the highest AUC score when applied to both SNV and INDEL variants.

Figure 2 summarized the performance of different filtering methods when applied to all SNV or INDEL variants (30× sequencing coverage). As it can be seen, when applied to the SNV variants identified by GATK, FVC scored the highest average AUC of 0.998, while the rest of the methods scored lower as follows: 0.989 (VEF), 0.785 (Frequency), 0.870 (Hard-Filter), 0.926 (VQSR), and 0.981 (GARFIELD). Concerning the INDEL variants, FVC exhibited even more improvements with an average AUC of 0.984. The rest of the methods scored lower as follows: 0.819 (VEF), 0.853 (Frequency), 0.733 (Hard-Filter), 0.836 (VQSR), and 0.783 (GARFIELD). When running with the default cut-off value of 0.5, FVC was not the best method of eliminating the highest number of false INDEL variants, but it exhibited the best performance in eliminating the total number of false variants (Table 1). The filtering improvements achieved by FVC were also observed when applied to the variants identified by Mutect2, Varscan2, and DeepVariant (Supplementary Tables 4–6). It is worth noting that FVC removed far more false variants than the current state-of-the-art methods in most cases, and it recalled ~51–99% true variants filtered out by the others. Moreover, FVC decreased the ratio of the eliminated true variants versus the removed false variants (OFO) from 0.05-1661.28 to 0.02-0.57 (Supplementary Table 7).

**Table 1:** The average performance of filtering methods when applied to the whole-genome sequencing datasets (HG001, HG003, HG004, and HG006) at 30× coverage and identified by GATK HaplotypeCaller.

Variant	Filter	AUC	AUPRG	MCC	OFO	F1-minor	BACC	NPV	TN	FN
SNV	FVC	0.998	0.92	0.89	0.08	0.89	0.93	0.93	5,869	425
	Frequency	0.785	0.33	0.50	1.54	0.49	0.79	0.45	3,902	5,046
	GARFIELD	0.981	0.35	0.28	10.49	0.19	0.86	0.11	5,119	42,888
	Hard-Filter	0.870	0.14	0.37	6.29	0.31	0.87	0.21	5,123	25,187
	VEF	0.989	0.87	0.84	0.17	0.84	0.91	0.86	5,627	894
	VQSR	0.926	0.05	0.18	24.96	0.09	0.86	0.05	5,121	112,859
INDEL	FVC	0.984	0.68	0.70	0.16	0.68	0.79	0.87	543	86
	Frequency	0.853	0.04	0.21	19.13	0.12	0.85	0.07	668	10,855
	GARFIELD	0.783	0.13	0.09	69.39	0.03	0.79	0.01	642	46,167
	Hard-Filter	0.733	0.02	0.14	22.98	0.08	0.73	0.04	477	10,636
	VEF	0.819	0.05	0.07	8.67	0.06	0.52	0.11	37	306
	VQSR	0.836	0.08	0.15	13.66	0.13	0.62	0.09	246	2,464

TN is the number of false variants that are filtered; FN is the number of true variants that are filtered. OFO equivalents to the ratio of the number of true variants that are eliminated (FN) versus the number of false variants that are removed (TN).

**Fig. 3:**
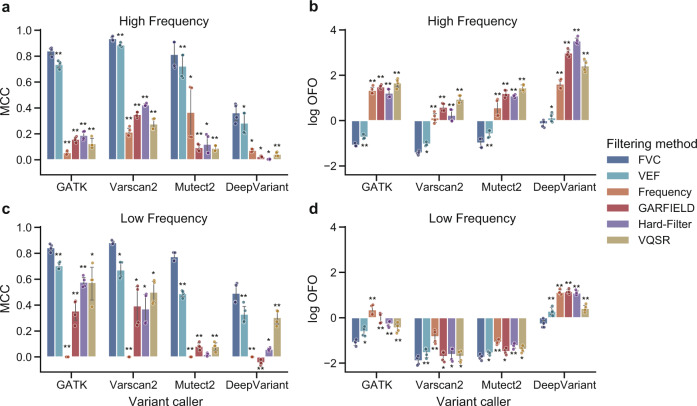
The performance of different filtering methods when applied to high-frequency variants or low-frequency variants. The high-frequency variants and low-frequency variants used as testing data are derived from whole-genome sequencing datasets (HG001, HG003, HG004, and HG006) at 30× coverage and identified by GATK, Varscan2, Mutect2, and DeepVariant, separately. Variant calls with variant allelic frequency (VAF) of more than 20% are defined as high-frequency variants. The other variant calls are defined as low-frequency variant calls. The performance of the filtering methods is assessed on the **a** high frequency variants using MCC; **b** high frequency variants using OFO; **c** low frequency variants using MCC; and **d** low frequency variants using OFO. The circle indicates the metric score achieved by FVC when applied to each specified testing data. The error bar indicates the 95% confidence intervals (*n* = 4 biologically independent samples). Asterisk denotes the significance of the comparison using a one-sided paired T-test (*p < 0.05, **p < 0.001), where the null hypothesis is that the FVC performs no better than the compared method. FVC consistently shows log OFO < 0 when applied to high-frequency and low-frequency variants.

Figure 3 summarized the performance of different filtering methods when applied to high-frequency (VAF ≥ 20%) or low-frequency (VAF < 20%) variants (30× sequencing coverage). When running FVC with the default cut-off value 0.5 and running other filtering methods with their suggested criteria, FVC also exhibited significant improvements than the other five filtering methods in the two subgroup variants, reflected by the highest MCC score and the lowest OFO score (*p* < 0.05, one-sided paired T-test). All filtering methods except VEF demonstrated better performance when applied to the low-frequency variants than high-frequency ones.

**Fig. 4:**
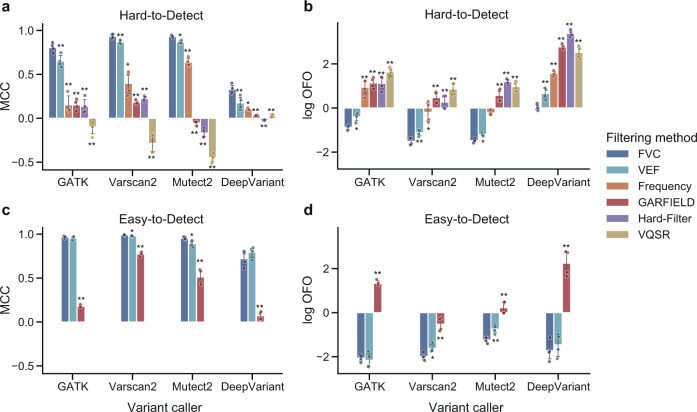
The performance of different filtering methods when applied to hard-to-detect or easy-to-detect variants. The easy-to-detect and hard-to-detect variant calls used as testing data are derived from the whole-genome sequencing datasets (HG001, HG003, HG004, and HG006) at 30× coverage and identified by GATK, Varscan2, Mutect2, and DeepVariant, separately. The easy-to-detect variants are defined as the variants that are consistently and correctly classified by all the unsupervised filtering methods (Frequency, Hard-Filter, and VQSR). The other variants are defined as hard-to-detect variants. The performance of the filtering methods is assessed on the **a** hard-to-detect variants using MCC; **b** hard-to-detect variants using OFO; **c** easy-to-detect variants using MCC (Frequency, Hard-Filter, and VQSR consistently scored MCC = 1); and **d** easy-to-detect variants using OFO (Frequency, Hard-Filter, and VQSR consistently scored logOFO = −∞). The circle indicates the metric score achieved by the filtering method when applied to each specified testing data. The error bar indicates the 95% confidence intervals (*n* = 4 biologically independent samples). Asterisk denotes the significance of the comparison using a one-sided paired T-test (*p < 0.05, **p < 0.001), where the null hypothesis is that the FVC performs no better than the compared method.

Figure 4 summarized the performance of different filtering methods when applied to easy-to-detect or hard-to-detect variants (30× sequencing coverage). The easy-to-detect variants are defined as the variants that are consistently and correctly classified by the three unsupervised methods (Frequency, Hard-Filter, VQSR), the others are defined as hard-to-detect variants that are incorrectly classified by at least one of the three methods. As can be observed, FVC exhibited superior performance when applied to the hard-to-detect variants in all cases. With respect to the easy-to-detect variants identified by Mutect2 and Varscan2, FVC also achieved significant improvements, reflected by the highest MCC and the lowest OFO (*p* < 0.05, one-sided paired T-test). When assessing on the easy-to-detect variants identified by DeepVariant, VEF scored the highest MCC but FVC achieved the lowest OFO. Both FVC and VEF exhibited better performance than GARFIELD in all cases.

**Fig. 5:**
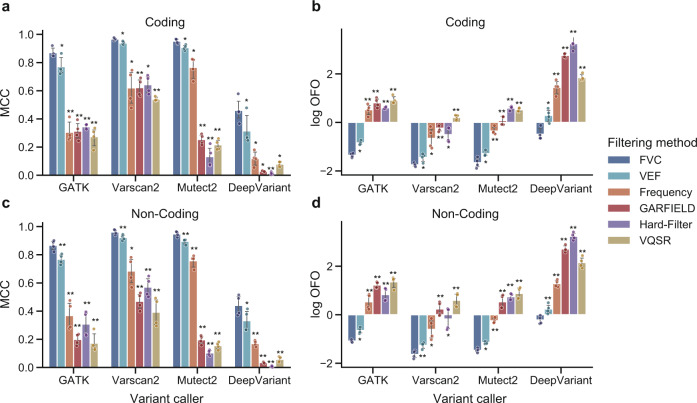
The performance of different filtering methods when applied to coding or non-coding variants. The coding and non-coding variant calls used as testing data are derived from whole-genome sequencing data (HG001, HG003, HG004, and HG006) at 30× coverage and identified by GATK, Varscan2, Mutect2, and DeepVariant, separately. The different filtering methods are separately assessed on the **a** coding variants using MCC; **b** coding variants measured using OFO; **c** non-coding variants measured using MCC; and **d** non-coding variants using OFO. FVC achieves the highest MCC and the lowest log OFO when applied to both types of variants identified by GATK HaplotypeCaller, Varscan2, Mutect2, and DeepVariant. The circle indicates the metric score achieved by the filtering method when applied to each specified testing data. The error bar indicates the 95% confidence intervals (*n* = 4 biologically independent samples). Asterisk denotes the significance of the comparison using a one-sided paired T-test (*p < 0.05, **p < 0.001), where the null hypothesis is that the FVC performs no better than the compared method.

Figure 5 summarized the performance of different filtering methods when applied to coding or non-coding variants. We could find that FVC not only achieved the highest MCC score both in coding and non-coding variants but was also the only method that consistently obtained OFO < 1 in these two types of variants.

We then assessed the performance of different filtering methods on an additional WGS dataset (HG007) which was not used in the above experiments. In this experiment, the pre-trained FVC and VEF were built on the training variants derived from four individuals (HG001, HG003, HG004, and HG006). As shown in Supplementary Fig 4, FVC consistently achieved the highest AUC score on SNV and INDEL variants. The improvements can also be observed when assessing with the corresponding area under the precision-recall-gain curves (AUPRG)^37^, the MCC score, the accuracy (ACC), the balanced accuracy (BACC), and the OFO (Supplementary Data 2).

The different filtering methods were also assessed using the leave-one-chromosome-out cross-validation method to remove possible bias. Specifically, we used the autosome variants derived from five human samples (HG001, HG003, HG004, HG006, and HG007). Sampling was implemented on the 22 chromosomes 22 times. Each time, a different chromosome was left out, the genetic variants from the left chromosome formed the test data, and the others formed the training data. There is no duplication between the training and testing data. Consistent with the leave-one-individual-out cross-validation measurement results, FVC achieved the highest AUC scores when applied to SNV and INDEL variants (Supplementary Fig. 5).

To further validate the classification performance of FVC, the above comparisons were also performed on the sequencing data at 50× coverage. In particular, the pre-trained models of FVC and VEF built on 30× coverage data were applied to the data at 50× coverage to test their generalization ability. Similar improvements of FVC were also observed in all cases (Supplementary Data 2–4).

## Discussion

Herein, with the goal to adapt different variant callers and address the fact that a large number of variant calls are misclassified by current filtering methods, especially in the case of low-frequency or hard-to-detect SNV and INDEL variants, we presented FVC—an adaptive method for quality control of variant calls, and demonstrated the improved performance both in minimizing the eliminating of true variants and maximizing the removal of false variants.

In most cases, the filtering method developed for a specific variant caller cannot be applied to other variant callers. It is partly because the development of filtering methods relies on the features generated by a specific variant caller. However, for the same variation, different variant callers will generate different numbers and types of features, making the filtering method unable to be used in different variant callers. To solve this problem, we developed a data construction module to build consistent features for the variants from different variant callers. Ideally, it can be immediately integrated into any variant calling pipeline. However, though we have tested its ability on four widely used variant calling pipelines, limitations still exist when the specific pipeline doesn’t provide the variants in VCF format.

Additionally, we found that the FVC models using the imbalanced training data outperformed those using balanced ones, which is different from the previous work^38^. This aspect is partly due to the fact that the ratio of the two classes in our study is up to 2204:1, which is much higher than the imbalance ratio of 10:1 in the previous study, and the number of samples (millions) used in this study is far more than the number of samples (thousands) in the study mentioned above.

Concerning the adaptive ability, we have provided four pre-trained models for filtering variants detected by GATK, Mutect2, Varscan2, and DeepVariant. However, the needs of users are not limited to the four variant callers. In case the user’s goal is to filter variants identified by a different pipeline, such as Pindel^39^ or Strelka2^40^, an adaptive filtering model could be built by providing three or four gold standard samples’ variants identified by the variant caller. It should be noted that the same model features, such as the median base quality (MBQ) or the variant allelic frequency (AF), showed different rankings of feature importance in different pre-trained models (Supplementary Fig 6). Hence, we suggest that the pre-trained model should be used on the variants derived from the same caller.

FVC was developed and assessed on the sequencing data consisting of 150bp and 250bp paired-end reads, and exhibited excellent performance on the variant calls derived from sequencing data at 30× coverage and 50× coverage. However, other usage scenarios of FVC, such as on data produced by utilizing different sequencing libraries (such as 2 × 300bp pair-end sequencing), different sequencing machines (such as Hiseq X), or different sequencing coverages (such as 10×), are feasible. In such cases, an adaptive pre-trained model could also be built by providing the gold standard samples’ variants derived from a public database or private sequencing data. The main limitation of FVC is that it focuses on the application in a single sample, and the information from other samples is not incorporated into FVC. For this case, other methods such as VQSR and ForestQC may be preferred, but FVC also supports users splitting the VCF file with multiple samples into multiple VCF files with a single sample and then performing quality control independently.

Considering that the comparisons in this study are all measured on the germline variants, the analytical performance may differ in tumor samples as somatic variants are often at lower than 20% variant allele frequency^41^. However, we find that FVC achieved similar performance when applied to the low-frequency variants and the high-frequency variants in some circumstances. For example, when applied to the variants detected by GATK HaplotypeCaller, FVC achieved similar MCC scores between high-frequency variants (0.841) and low-frequency variants (0.844). When applied to the low-frequency variants detected by Mutect2 and Varscan2, FVC exhibited slightly lower MCC scores within 0.06 on average. Thus, it may imply that FVC could perform well for somatic variant filtration when enough gold-standard somatic variant calls become available for building a pre-trained model.

Furthermore, FVC could also be helpful for post-filtering of RNA-sequencing mutation detection pipelines as they are similar with the DNA-sequencing mutation detection pipelines^20,42^, such as performing read alignment by using the BWA or other alignment tools and detecting variations by using GATK, SNPiR^43^ or other variant callers^44,45^.

Users should comprehensively consider the evaluation results of multiple metrics when deciding which software to use. In this study, we performed the comparisons by using sixteen metrics. FVC does not perform well on all evaluation metrics (Supplementary Data 3 and 4). Actually, these filtering methods exhibited little difference in the F1-major score, sensitivity, and specificity. For example, we can find that all filtering methods scored F1-major score > 0.96, sensitivity > 0.93, and specificity > 0.998 when applied to GATK detected variants in HG001 sample, the recall was decreased and the precision was improved after filtering by any one of the filtering methods (Supplementary Data 3).

However, when assessing with the newly defined metric OFO, the filtering methods exhibited extremely different performance. For example, when applied to the GATK detected variants from WGS Human sample HG001 (Supplementary Table 8), the Hard-Filter eliminated 24.06 true INDELs and 9.83 true SNVs per removing one false variant correspondingly. FVC exhibited not extremely different with the Hard-Filter in recall, precision, and F1-major score. But it decreased the loss of true variants to 0.16 and 0.08 per filtering one false INDEL and one false SNV, respectively. Though the Hard-Filter achieved similar F1-major score with FVC, the presented FVC method demonstrated more suitable in the filtering of GATK detected variants. Therefore, in the choosing of which filter to use, users should make further decisions based on OFO and other comprehensive metrics, such as AUC and MCC, when the objective metrics achieved similar scores.

Taking it all together, FVC presented a superior performance in the accuracy, generalization ability, application scope, which could potentially be used to integrate variant calls detected by multiple variant callers.

## Methods

### Data preparation

Whole-genome sequencing data from five individuals were used in this study: one pilot genome HG001/NA12878 from the HapMap project^46^; two Ashkenazim individuals—HG003/NA24149 and HG004/NA24143; and two Chinese individuals—HG006/NA24694 and HG007/NA24695. There is no consanguinity between these five samples and all these sequencing data were released by NIST’s GIAB consortium^32,35^. The downloaded whole-genome sequencing datasets were generated on Illumina HiSeq 2500 platform (Illumina Inc, San Diego, USA) with 2 × 148bp (HG001, HG006, HG007) or 2 × 250bp (HG003 and HG004) paired-end reads. The mean coverage of the sequencing data ranged from 50× to 300×. The source of the dataset is listed in the Supplementary Method.

Firstly, the downloaded sequencing data was realigned to human genome build GRCh37 with the same pipeline. The sequencing reads were firstly randomly downsampled to ~30× and ~50× coverage by using Samtools^47^. Such levels are commonly used in WGS studies^48–50^, and few uncovered or uncalled bases above these depths^51^. Then, the reads in FASTQ format were realigned and converted to BAM format by performing sequencing realignment, marking duplicates, and local realignment using the BWA-MEM, Dedup, and Realigner software which were integrated into Sentieon's DNASeq^52^ pipeline.

Then, the genetic variants were derived by GATK HaplotypeCaller (version 4.0.11, with default parameters), Mutect2 (integrated in GATK version 4.1.9 with default parameters), Varscan2 (version 2.3.9 with default parameters, except where parameters–min-coverage 3,–p-value 0.10,–min-var-freq 0.01 were used), and DeepVariant (version 1.2, with default parameters). All variants were stored in variant call format (VCF) files.

Finally, true variants and false variants in the VCF files were labeled based on whether the variants were contained in the GIAB’s gold-standard variants or not with the help of RTG-vcfeval^53^ software (version 3.10 with parameters ‘squash-ploidy’ to ignore the zygosity differences). The resultant ‘true’ and ’false’ label-containing genetic variants from the four individuals (HG001, HG003, HG004, and HG006) were then utilized downstream in the leave-one-individual-out cross-validation assessment. The remainder variants from the HG007 sample were used as independent testing data. The true and false variants distributions in different subgroups are listed in Supplementary Table 9 and Supplementary Data 5.

### FVC feature construction module

A total of 20 features associated with each variant are selected or constructed by the FVC feature construction module. Firstly, FVC adds features for each genetic variant in the raw input VCF file by using GATK VariantAnnotator (version 4.1.9). Then, FVC selects and constructs features based on the variant position, variant types (SNV or INDEL), INFO column, and FORMAT column in the aforementioned processed VCF file. All the constructed features can be classified into three categories: sequence-related features (*n* = 4), experiment-related features (*n* = 8), and analysis-related features (*n* = 8). The definitions of each feature can be found in the Supplementary Method. The features in raw VCF and the features built by the FVC feature construction module are listed in Supplementary Table 10.

### FVC data construction module

In the data construction module, the RTG vcfeval software and the imbalanced method are introduced to construct training and testing data. Firstly, all the variants derived from four individuals (HG001, HG003, HG004, and HG006) are labeled as true or false genetic variants by using RTG vcfeval software according to comparison results with the gold standard variants. Then, the variants from three of four individuals are combined as the training data without balancing the two categories (true and false variants) in the leave-one-individual-out cross-validation step, and the variants from the left individual are regarded as the testing data. The genetic variants with the same chromosome, genome position, reference allele, alternative allele, features, and categories (true variant or false variant) are regarded as duplicates and are removed from the training data if they are also included in the test data in case of data leakage.

### FVC supervised learning and filtering module

FVC applies the XGBoost as the supervised learning algorithm and models the technical profile of true and false variants based on the features and labels built by the feature construction module and data construction module. Once trained, the FVC can be utilized to filter the variants identified from the same pipeline. In the filtering results, FVC provides a self-defined score in INFO field to indicate the probability of the variant being correct and specifies ‘Filtered’ in the FILTER field in VCF if the score is less than 0.5. The default parameters settings of XGBoost and the other candidate machine learning methods are listed in Supplementary Table 11.

### Performance analysis and dealing with data leakage

We compared the FVC with five other methods when applied to the WGS data. FVC used by default a threshold of 0.5, i.e., the genetic variant is regarded as a false variant if its probability of being correct is less than 0.5. The false variants defined by the other five methods were based on their suggested criteria (Supplementary Method).

The filtering methods were assessed in a head-to-head comparison using the leave-one-individual-out cross-validation method. For the assessment, we used the variant calls derived from 4 human samples (HG001, HG003, HG004, and HG006) and performed 4 subgroup analysis, including: 1) Assessing on SNV and INDEL variants, separately; 2) Assessing on high-frequency (VAF ≥ 20%) and low-frequency (VAF < 20%) variants, separately; 3) Assessing on hard-to-detect and easy-to-detect variants separately; 4) Assessing on coding and non-coding variants, separately.

To deal with the potential data leakage and bias, the same variant calls were removed from the training data if they also appeared in the testing data. Moreover, we compared the filtering methods on an additional variant call-set derived from the human sample HG007 which is not genetically related to the other individuals. Subsequently, the performance of the different filtering methods was further assessed using the leave-one-chromosome-out cross-validation method to ensure that there is no duplicate between the training and testing data. As not all gold-standard variant call-sets contain sexual chromosomes, the variants located on the autosomes were used in the cross-validation assessment. Specifically, sampling was implemented on the dataset 22 times. Each time a different chromosome was left out, the variant calls from the left chromosome formed the test dataset, and the others formed the training dataset.

### Evaluation metrics

Sixteen evaluation metrics were utilized to assess the performance of these different filtering methods. Three of them were used to assess the comprehensive performance under different thresholds, including the area under the receiver characteristic operator curve (AUC), the area under the precision-recall-gain curve (AUPRG), and the area under the precision-recall curve (AUPRC). It is worth noting that the outputs of Frequency and Hard-Filter were dichotomous. Therefore, we calculate these metrics in the two cases using the curve with three points: (0, y_1_), (x_2_, y_2_), (1, y_3_), where x and y are the values corresponding to the axes. For example, in calculating the AUC, y_1_ is the value of TPR (i.e., 0) when FPR = 0, x_2_ and y_2_ are the values of FPR and TPR using the suggested thresholds, respectively, y_3_ is the value of TPR (i.e., 1) when FPR = 1.

Four of these metrics were used to demonstrate the count of variants that were correctly or incorrectly retained or filtered by using the particular filtering method. True Positive (TP): the number of true variants that were retained; False Positive (FP): the number of false variants that were retained; False Negative (FN): The number of true variants that were eliminated; True Negative (TN): The number of false variants that were eliminated.

Seven metrics were used to demonstrate the comprehensive performance under the suggested threshold, including: F1 score for the majority class (F1-major); F1 score for the minority class (F1-minor); Matthews Correlation Coefficient (MCC); Accuracy (ACC); Balanced Accuracy (BACC); Precision; Sensitivity or named as True Positive Rate (TPR); Specificity or named as True Negative Rate (TNR).

The proportion of the false variants in the filtered variants can be assessed by Negative Predictive Value (NPV) in Eq. (1). However, one of the motivations in the filtering task is to minimize the number of true variants and maximize the number of false variants in the eliminated variant set (the predictive negative class). The NPV is necessary but cannot be used for this purpose. Therefore, we introduced the odds of false omission in the predicted negative class to intuitively describe the proportion of true and false variants in the eliminated variant set, which was abbreviated to OFO in this study (Eq. (2)). It is equivalent to the ratio of the number of eliminated true variants (FN) versus the number of eliminated false variants (TN).

1 $${{{{{\rm{NPV}}}}}}=\frac{{{{{{\rm{TN}}}}}}}{{{{{{\rm{TN}}}}}}+{{{{{\rm{FN}}}}}}}$$

2 $${{{{{\rm{OFO}}}}}}=\frac{{{{{{\rm{FOR}}}}}}}{1-{{{{{\rm{FOR}}}}}}}=\frac{{{{{{\rm{FOR}}}}}}}{{{{{{\rm{NPV}}}}}}}=\frac{{{{{{\rm{FN}}}}}}}{{{{{{\rm{TN}}}}}}}$$

Here, the false omission rate (FOR) = $\frac{{{{{{\rm{FN}}}}}}}{{{{{{\rm{TN}}}}}}\,+\,{{{{{\rm{FN}}}}}}}$. OFO ranges from 0 to +∞. When OFO = 1, the number of eliminated false variants is equal to the number of eliminated true variants. When OFO = 0, it means that there is no true variant in the eliminated variants, which seems perfect. +∞ occurs when FN>>TN, i.e., the number of true variants far exceeds the number of false variants in the filtered variant sets, which indicates the worst performance. In some circumstances, the filtering performance may be overestimated with OFO = 0. For example, if the filtering method eliminates only one genetic variant, and it is the false variant, i.e., the FN = 0, and the TN = 1, the filtering method will be overestimated with OFO = 0. Therefore, similar to sensitivity and specificity, OFO cannot be used alone to measure the model's comprehensive performance.

### Statistics and reproducibility

One-sided paired T-test was applied for pairwise group comparisons where the null hypothesis was that FVC performed no better than the compared filtering method. A *p* < 0.05 was considered statistically significant. *p < 0.05, **p < 0.001. All replicate experiments were performed using the leave-one-out cross-validation method. The statistical analysis and plotting were completed using the scikit-learn library in Python (version 3.6).

### Reporting summary

Further information on research design is available in the Nature Research Reporting Summary linked to this article.

## Supplementary information

Supplementary Information

Description of Additional Supplementary Files

Supplementary Data 1

Supplementary Data 2

Supplementary Data 3

Supplementary Data 4

Supplementary Data 5

Reporting Summary

## Data Availability

The raw data that support the findings of this study are publicly available in NIST’s GIAB repository (https://github.com/genome-in-a-bottle/giab_data_indexes/tree/master)^54^. The processed data that support the findings of this study are committed on the Dryad Digital Repository (10.5061/dryad.hdr7sqvkm)^55^. The source data underlying the graphs are provided within Supplementary Data files 1–5 (excel).

## Code availability

FVC is implemented in Perl (version 5.0) and Python (version 3.6), and the source code is publicly available at https://github.com/yyren/FVC and Zenodo (10.5281/zenodo.6379296)^56^.
