# Supplementary material for: FVC as an adaptive and accurate method for filtering variants from popular NGS analysis pipelines
Source: Commun Biol. 2022 Sep 16;5:975. doi: 10.1038/s42003-022-03397-7 (PMC9481582; doi:10.1038/s42003-022-03397-7)
Supplement: Supplementary file 1 — Supplementary Information [file 42003_2022_3397_MOESM1_ESM.pdf]

FVC as an adaptive and accurate method for filtering variants from popular NGS analysis pipelines

1. Supplementary Results

Nine of sixteen evaluation metrics are listed in the Supplementary Table 1-3. They are: the area under the ROC curve; the area under the precision-recall-gain curve (AUPRG); the Matthews Correlation Coefficient (MCC); the odds of false omission in the predict negative class (eliminated variants), named as OFO in this study, and it equivalents to the ratio of the true variants that were eliminated (FN) versus the number of false variants that were eliminated (TN); the F1 score for minority class (F1-minor); the Balanced Accuracy (BACC); the negative predictive value (NPV)= $\frac{TN}{TN+FN}$.

Supplementary Table 1. The average performance of FVC containing the raw VCF features or the constructed features when applied on the whole-genome sequencing datasets (HG001, HG003, HG004, and HG006) at 30x coverage.

Caller	Variant	Features	MCC	AUC	AUPRG	OFO	F1-minor	BACC	NPV	TN	FN
GATK	SNV	Constructed features	0.89	0.998	0.92	0.08	0.89	0.92	0.93	5870	425
		Raw VCF features	0.77	0.994	0.80	0.18	0.76	0.85	0.85	4872	840
	INDEL	Constructed features	0.70	0.984	0.68	0.16	0.68	0.79	0.86	546	88
		Raw VCF features	0.43	0.917	0.37	0.42	0.38	0.63	0.71	253	103
Mutect2	SNV	Constructed features	0.96	0.999	0.97	0.03	0.96	0.97	0.97	55661	1614
		Raw VCF features	0.94	0.994	0.96	0.04	0.94	0.96	0.96	54669	2260
	INDEL	Constructed features	0.89	0.993	0.91	0.06	0.89	0.92	0.94	6579	394
		Raw VCF features	0.85	0.989	0.88	0.09	0.85	0.89	0.92	6255	497
Varscan2	SNV	Constructed features	0.97	0.999	0.986	0.02	0.97	0.98	0.98	51999	970
	INDEL	Constructed features	0.83	0.986	0.828	0.09	0.82	0.87	0.92	2030	149
DeepVariant	SNV	Constructed features	0.48	0.989	0.461	0.62	0.46	0.68	0.63	414	205
	INDEL	Constructed features	0.35	0.982	0.353	0.76	0.31	0.61	0.57	147	113

The features were tested on the FVC employing the same data construction module (imbalanced data) and supervised learning module (employing XGBoost).

Supplementary Table 2. The performance of FVC employing different data construction methods when applied on the whole-genome sequencing datasets (HG001, HG003, HG004, and HG006) at 30x coverage and identified by GATK HaplotypeCaller.

Method	Variant	MCC	AUC	AUPRG	OFO	F1-minor	BACC	NPV	TN	FN
FVC module	SNV	**0.89**	0.998	0.92	0.08	0.89	0.92	0.93	5870	425
	INDEL	**0.70**	0.984	0.68	0.16	0.68	0.79	0.86	546	88
SMOTE	SNV	0.87	0.997	0.91	0.17	0.87	0.94	0.85	6082	1020
	INDEL	0.53	0.977	0.56	1.27	0.52	0.82	0.45	599	709
Random upsampling	SNV	0.65	0.995	0.89	1.39	0.60	0.97	0.45	6460	7929
	INDEL	0.47	0.970	0.61	2.37	0.43	0.87	0.30	692	1575
Random undersampling	SNV	0.39	0.997	0.86	5.92	0.27	0.98	0.16	6679	36159
	INDEL	0.20	0.983	0.44	22.71	0.08	0.94	0.04	855	18691
NearMiss-1	SNV	0.04	0.961	0.50	337.69	0.01	0.70	0.00	6729	1831180
	INDEL	-0.03	0.518	0.00	554.44	0.00	0.48	0.00	865	440255

The different data construction methods were tested on the FVC employing the same features construction module (constructed features) and supervised learning module (employing XGBoost).

Supplementary Table 3. The performance of FVC employing different supervised learning methods when applied on the whole-genome sequencing datasets (HG001, HG003, HG004, and HG006) at 30x coverage and identified by GATK HaplotypeCaller.

Method	Variant	MCC	AUC	AUPRG	OFO	F1-minor	BACC	NPV	TN	FN
XGBoost	SNV	**0.89**	0.998	0.92	0.08	0.89	0.92	0.93	5870	425
	INDEL	**0.70**	0.984	0.68	0.16	0.68	0.79	0.86	546	88
Random Forest	SNV	0.88	0.984	0.92	0.07	0.88	0.92	0.94	5755	392
	INDEL	0.68	0.945	0.68	0.13	0.66	0.77	0.88	504	67
MLP	SNV	0.83	0.998	0.89	0.09	0.83	0.88	0.92	5325	489
	INDEL	0.57	0.983	0.56	0.26	0.53	0.71	0.79	413	107
Logistic Regression	SNV	0.79	0.996	0.82	0.18	0.79	0.86	0.85	5067	747
	INDEL	0.43	0.950	0.38	0.39	0.37	0.63	0.73	239	80
Linear SVM	SNV	0.77	0.996	0.81	0.21	0.77	0.86	0.84	4893	819
	INDEL	0.16	0.939	0.37	0.19	0.07	0.52	0.85	28	6
LightGBM	SNV	0.73	0.878	0.72	0.37	0.72	0.86	0.73	5005	1729
	INDEL	0.44	0.837	0.36	1.61	0.44	0.75	0.40	478	851

MLP: Multilayer Perceptron Neural Network; The different supervised learning methods were tested on the FVC employing the same features construction module (constructed features) and data construction module (construct imbalanced set).

Supplementary Table 4. The performance of filtering methods when applied on the whole-genome sequencing datasets (HG001, HG003, HG004, and HG006) at 30x coverage and identified by Mutect2.

Variant	Filter	AUC	AUPRG	MCC	OFO	F1-minor	BACC	NPV	TN	FN
SNV	FVC	**0.999**	**0.97**	**0.96**	**0.03**	**0.96**	0.97	**0.97**	**55661**	**1614**
	Frequency	0.951	0.78	0.83	0.32	0.83	0.95	0.76	54153	15527
	GARFIELD	0.670	0.14	0.21	3.90	0.21	0.61	0.24	11793	39229
	Hard-Filter	0.549	0.06	0.13	4.49	0.12	0.55	0.21	4858	21590
	VEF	0.994	0.96	0.94	0.06	0.94	0.97	0.94	55531	3628
	VQSR	0.911	0.08	0.16	8.58	0.16	0.63	0.12	15723	115715
INDEL	FVC	0.993	0.91	0.89	0.06	0.89	0.92	0.94	6579	394
	Frequency	0.944	0.21	0.52	2.37	0.45	0.94	0.30	7279	18460
	GARFIELD	0.601	0.08	0.07	5.82	0.05	0.51	0.23	216	962
	Hard-Filter	0.506	0.01	0.01	35.16	0.03	0.51	0.03	190	6870
	VEF	0.963	0.55	0.47	0.26	0.41	0.64	0.80	2096	458
	VQSR	0.769	0.10	0.15	3.60	0.14	0.55	0.25	807	2378

Supplementary Table 5. The performance of filtering methods when applied on the whole-genome sequencing datasets (HG001, HG003, HG004, and HG006) at 30x coverage and identified by Varscan2.

Variant	Filter	AUC	AUPRG	MCC	OFO	F1-minor	BACC	NPV	TN	FN
SNV	FVC	**0.999**	**0.99**	**0.97**	**0.02**	**0.97**	**0.98**	**0.98**	**51999**	**970**
	Frequency	0.804	0.58	0.72	0.17	0.71	0.80	0.87	33876	3665
	GARFIELD	0.959	0.39	0.48	1.97	0.46	0.83	0.38	33907	55959
	Hard-Filter	0.811	0.41	0.59	0.86	0.58	0.81	0.61	31042	20788
	VEF	0.998	0.98	0.95	0.06	0.95	0.97	0.95	51380	2618
	VQSR	0.962	0.14	0.40	4.57	0.34	0.89	0.22	44613	157729
INDEL	FVC	**0.986**	**0.83**	**0.83**	**0.09**	**0.82**	**0.87**	**0.92**	**2030**	149
	Frequency	0.861	0.26	0.47	2.94	0.43	0.86	0.32	1952	4399
	GARFIELD	0.860	0.16	0.21	4.00	0.20	0.59	0.27	462	1396
	Hard-Filter	0.684	0.19	0.30	5.83	0.30	0.68	0.26	1118	3296
	VEF	0.835	0.08	0.04	1.95	0.01	0.50	0.41	10	14
	VQSR	0.886	0.15	0.19	3.74	0.19	0.58	0.24	423	1323

Supplementary Table 6. The performance of filtering methods when applied on the whole-genome sequencing datasets (HG001, HG003, HG004, and HG006) at 30x coverage and identified by DeepVariant.

Variant	Filter	AUC	AUPRG	MCC	OFO	F1-minor	BACC	NPV	TN	FN
SNV	FVC	0.989	0.46	0.48	0.62	0.46	0.68	0.634	414	205
	Frequency	0.765	0.06	0.23	10.92	0.17	0.77	0.106	575	5521
	GARFIELD	0.915	0.05	0.04	408.10	0.01	0.83	0.003	807	276136
	Hard-Filter	0.615	0.00	0.01	2407.04	0.00	0.62	0.000	1088	2324164
	VEF	0.982	0.40	0.45	1.21	0.45	0.72	0.476	477	486
	VQSR	0.969	0. 00	0.07	164.97	0.01	0.86	0.007	824	115588
INDEL	FVC	0.982	0.35	0.35	0.76	0.31	0.61	0.571	147	113
	Frequency	0.825	0.02	0.14	32.21	0.06	0.83	0.030	458	14540
	GARFIELD	0.755	0.02	-0.01	709.08	0.00	0.49	0.001	652	447016
	Hard-Filter	0.487	0.00	-0.01	719.79	0.00	0.49	0.001	541	382061
	VEF	0.929	0.07	0.10	6.98	0.10	0.543	0.128	56	380
	VQSR	0.888	0.02	0.05	36.59	0.04	0.538	0.030	55	1720

Supplementary Table 7. The performance of filtering methods when applied on the whole-genome sequencing datasets (HG001, HG003, HG004, and HG006) at 30x coverage.

Variant Caller	Filtering software	OFO	TN	FN	Recalled by FVC
GATK	FVC	0.08	**6,412**	511	-
	Frequency	3.48	4,570	15,901	97%
	GARFIELD	15.46	5,761	89,055	99%
	Hard-Filter	6.40	5,600	35,823	99%
	VEF	0.21	5,664	1,200	57%
	VQSR	21.49	5,367	115,323	99%
Mutect2	FVC	0.03	**62,240**	2,008	-
	Frequency	0.55	61,432	33,987	94%
	GARFIELD	3.35	12,009	40,191	95%
	Hard-Filter	5.64	5,048	28,460	93%
	VEF	0.07	57,627	4,086	**51%**
	VQSR	7.14	16,530	118,093	98%
Varscan2	FVC	0.02	**54,029**	1,119	
	Frequency	0.23	35,828	8,064	86%
	GARFIELD	1.67	34,369	57,355	98%
	Hard-Filter	0.75	32,160	24,084	95%
	VEF	0.05	51,390	2,632	57%
	VQSR	3.53	45,036	159,052	99%
DeepVariant	FVC	0.57	561	318	-
	Frequency	19.42	1,033	20,061	98%

	GARFIELD	495.65	1,459	723,152	99%
	Hard-Filter	1661.28	1,629	2,706,225	**99%**
	VEF	1.62	533	866	63%
	VQSR	133.46	879	117,308	99%

Recalled by FVC: the proportion of eliminated true variants recalled by the FVC. FVC recalled ~51-99% true variants filtered out by the others. Moreover, FVC decreased the ratio of the eliminated true variants versus the removed false variants (OFO) from 0.05 (VEF on Varscan2 detected variants) - 1661.28 (Hard-Filter on DeepVariant detected variants) to 0.02 (FVC on Varscan2 variants) - 0.57 (FVC on DeepVariant detected variants).

Supplementary Table 8. The performance of filtering methods when applied to the GATK detected variants from WGS Human sample HG001 (30× coverage).

Variant	Pipeline	TP	FP		FN		Sensitivity	Specificity	F1-major	**OFO**
			FP_{vcf}	TN_{filter}	$FN_{original}$	FN_{filter}				
SNV	Original	3207484	5402	-	2285	-	0.9993	0.9983	0.9988	-
	Original+ FVC	3207170	1258	4144	2285	314	0.9992	0.9996	0.9994	**0.08**
	Original+ VEF	3206807	1345	4057	2285	677	0.9991	0.9996	0.9993	0.17
	Original + Hard-Filter	3169309	1519	3883	2285	38175	0.9874	0.9995	0.9934	9.83
	Original+ Frequency	3199205	2172	3230	2285	8279	0.9967	0.9993	0.9980	2.56
	Original+ VQSR	3077810	1418	3984	2285	129674	0.9589	0.9995	0.9788	32.55
	Original+ GARFIELD	3154853	2169	3233	2285	52631	0.9829	0.9993	0.9910	16.28
INDEL	Original	478261	722	-	3539	-	0.9927	0.9985	0.9956	-
	Original+	478205	381	341	3539	56	0.9925	0.9992	0.9959	**0.16**

	FVC									
	Original+ VEF	477884	685	37	3539	377	0.9919	0.9986	0.9952	10.19
	Original+ Hard-Filter	471332	434	288	3539	6929	0.9783	0.9991	0.9886	24.06
	Original+ Frequency	460669	199	523	3539	17592	0.9561	0.9996	0.9774	33.64
	Original+ VQSR	475330	599	123	3539	2931	0.9866	0.9987	0.9926	23.83
	Original+ GARFIELD	450290	294	428	3539	27971	0.9346	0.9993	0.9659	65.35

The Original indicates the variants that are detected by the GATK. The $FN_{original}$ indicates the true variants that are not detected by the GATK. The $OFO=FN_{filter}/TN_{filter}$, where the FN_{filter} indicates the true variants in the original but are eliminated by the filtering method, the TN_{filter} indicates the false variants in the original but are removed by the filtering method.

Supplementary Figure 1 summarized the filtering accuracy of FVC with different input features – features in the raw input VCF file or the features constructed by the FVC module. The raw VCF features are the corresponding features in the INFO field of VCF file generated by GATK or Mutect2. The constructed features are the features in the INFO field of VCF file or constructed by FVC (Supplementary Table 9). As it can be observed, FVC containing the constructed features achieved significant improvements over the FVC containing the raw VCF features when applied to SNV and INDEL variants identified by GATK or Mutect2. Specifically, as shown in Supplementary Table 1, when applied to the variants identified by GATK, FVC containing the constructed features scored higher MCC values of 0.89 (SNV) and 0.70 (INDEL). In comparison, FVC containing the raw VCF features scored lower MCC values of 0.77 (SNV) and 0.43 (INDEL). The improved performance could also be observed when applied to the Mutect2 detected variants. Moreover, concerning the variants identified by Varscan2 and DeepVariant, the raw VCF feature set is empty, i.e., FVC could not be built using the raw VCF features. In these two cases, FVC containing the constructed features exhibited better performance when applied to Varscan2 detected variants than applied to DeepVariant detected

variants. The improvements achieved by incorporating constructed features were also observed when assessing with other evaluation metrics (Supplementary Data 1).

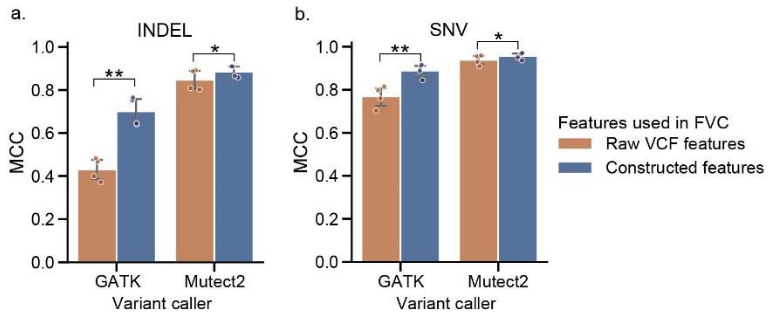

Supplementary Figure 1 Comparison results of the performance of FVC containing raw VCF features or containing constructed features. The SNV and INDEL variants used as testing data are derived from whole-genome sequencing datasets (HG001, HG003, HG004, and HG006) at 30× coverage and identified by GATK and Mutect2, separately. The performance of a machine learning method is affected by the variable feature space though it is trained on the same variant call set. The pre-trained FVC models containing two different sets of input features are assessed on the **a.** insertion and deletion (INDEL) variants; **b.** single nucleotide variants (SNV). The details of the construction of the two sets of features are listed in the Method and Supplementary Table 9. The performance is assessed by using the leave-one-individual-out cross-validation method. The circle indicates the MCC score achieved by FVC when applied to each specified testing data. The error bar indicates the 95% confidence intervals of MCC (n=4 biologically independent samples). Asterisk denotes the significance of the comparison using a one-sided paired T-test (*p<0.05, **p<0.001), where the null hypothesis is that the FVC containing the raw VCF features performs no better than the FVC containing the constructed features.

Supplementary Figure 2 summarized the filtering accuracy of FVC with different methods of constructing training data. The proposed FVC module achieved the best performance, which contains the method of constructing an imbalanced training set instead of a balanced training set. In more detail (Supplementary Table 2), when assessing on the variants identified by GATK, the proposed FVC module achieved the highest MCC scores of 0.89 (SNV) and 0.70 (INDEL). The SMOTE, constructing a balanced training set by interpolating the minority class, exhibited slightly lower MCC scores of 0.87 (SNV) and 0.53 (INDEL). The method for random upsampling of the minority class achieved the MCC scores of 0.65 (SNV) and 0.47 (INDEL). Nevertheless, these two upsampling methods exhibited better performance than the following two downsampling methods. The method for random downsampling of the majority class scored lower MCC scores of 0.39 (SNV) and 0.20 (INDEL). The prototype-selection-based

downsampling method NearMiss-1 exhibited the worst performance with the MCC scores of 0.04 (SNV) and -0.03 (INDEL). The improvements achieved by the method of constructing an imbalanced training set were also observed on the variants identified by Mutect2, Varscan2, and DeepVariant (Supplementary Data 1).

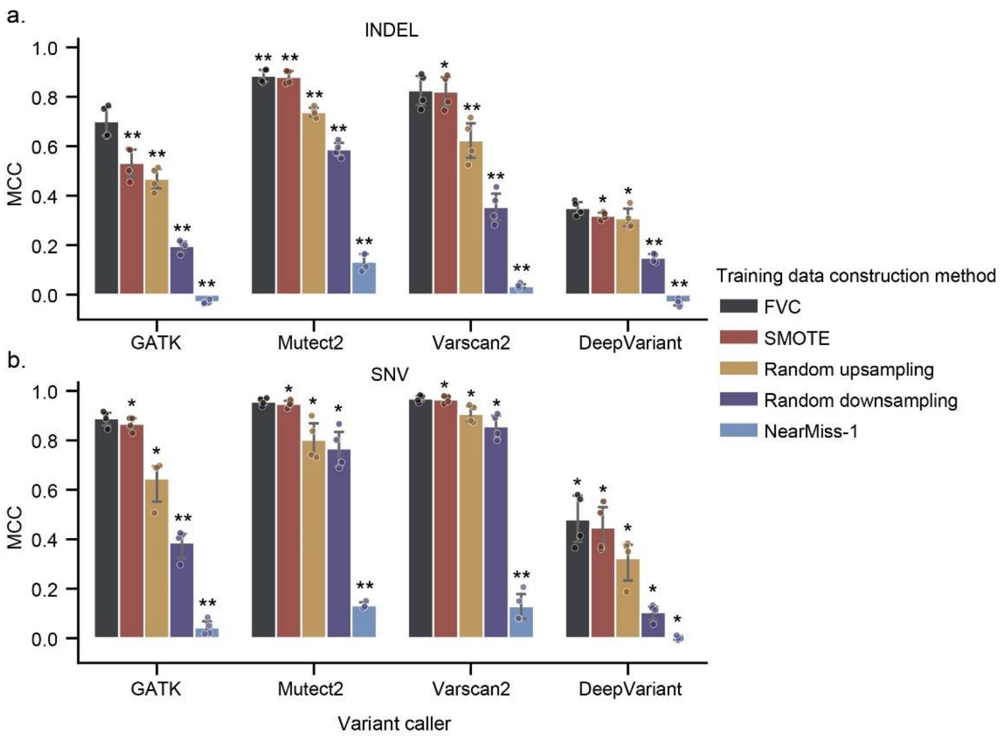

Supplementary Figure 2 The performance of FVC containing different training data construction methods. The SNV and INDEL variants used as testing data are derived from whole-genome sequencing datasets (HG001, HG003, HG004, and HG006) at 30× coverage and identified by GATK, Varscan2, Mutect2, and DeepVariant, separately. The filtering performance of FVC containing different training data construction methods is measured when applied to the **a.** insertion and deletion (INDEL) variants; **b.** single nucleotide variants (SNV). The performance is assessed by using the leave-one-individual-out cross-validation method. The circle indicates the MCC score achieved by FVC when applied to each specified testing data. The error bar indicates the 95% confidence intervals (n=4 biologically independent samples). Asterisk denotes the significance of the comparison using a one-sided paired T-test (*p<0.05, **p<0.001), where the null hypothesis is that the FVC trained on the data constructed by the imbalanced method (FVC data construction module) performs no better than the FVC trained on the data constructed with a balanced method.

The filtering accuracy of FVC incorporated with different machine learning methods was shown in Supplementary Figure 3. As it can be observed, FVC embedding XGBoost exhibited significantly improved classification ability over the other methods in most cases. In more detail (Supplementary Table 3), FVC embedding XGBoost scored the highest MCC scores of

0.89 (SNV) and 0.70 (INDEL) when assessing on the variants identified by GATK. In contrast, the scores of the other methods achieved lower MCC scores of 0.88 (SNV) and 0.68 (INDEL) in Random Forest; 0.83 (SNV) and 0.57 (INDEL) in Multilayer Perceptron Neural Network; 0.79 (SNV) and 0.43 (INDEL) in Logistic Regression; 0.77 (SNV) and 0.16 (INDEL) in Linear SVM; 0.73 (SNV) and 0.44 (INDEL) in LightGBM. The filtering improvements achieved by the XGBoost were also observed when applied to the variants identified by Mutect2, Varscan2, and DeepVariant (Supplementary Data 1).

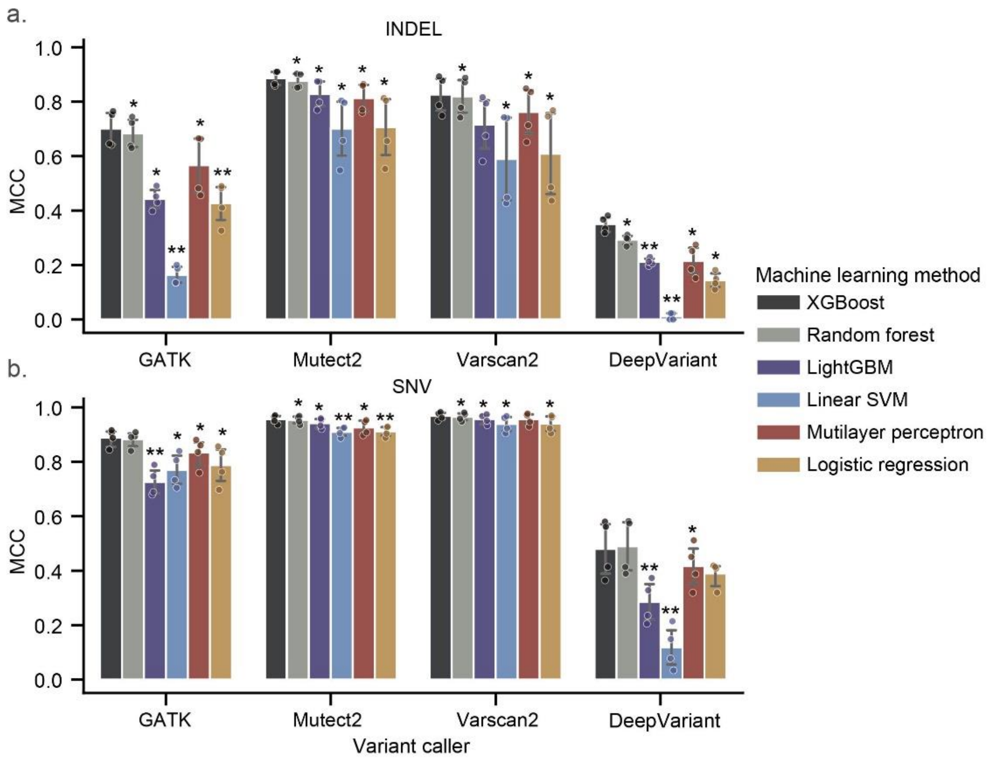

Supplementary Figure 3 Comparison of the performance of FVC using different machine learning methods. The SNV and INDEL variants used as testing data are derived from whole-genome sequencing datasets (HG001, HG003, HG004, and HG006) at 30× coverage and identified by GATK, Varscan2, Mutect2, and DeepVariant, separately. The filtering performance of FVC containing different supervised learning methods is assessed on the **a.** insertion and deletion (INDEL) variants; **b.** single nucleotide variants (SNV). The performance is assessed by using the leave-one-individual-out cross-validation method. The circle indicates the MCC score achieved by FVC when applied to each specified testing data. The error bar indicates the 95% confidence intervals (n=4 biologically independent samples). Asterisk denotes the significance of the comparison using a one-sided paired T-test (*p<0.05, **p<0.001), where the null hypothesis is that the XGBoost performs no better than the compared method.

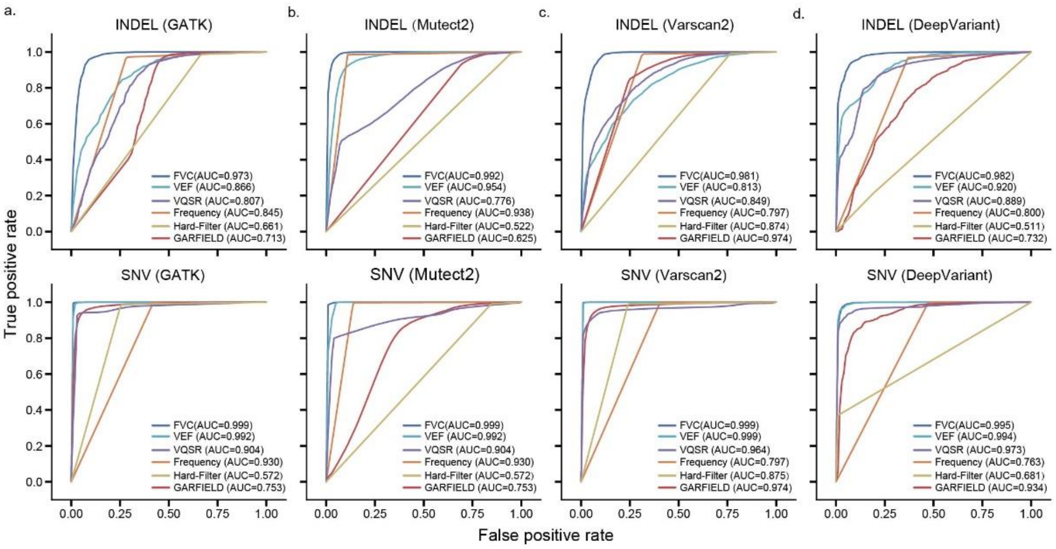

Supplementary Figure 4 The performance of different filtering methods when applied on HG007 sample. The SNV and INDEL variants used as testing data are derived from HG007 whole-genome sequencing data at 30x coverage. The performance of different filtering methods is assessed on the SNV and INDEL variants identified by **a.** GATK HaplotypeCaller; **b.** Mutect2; **c.** Varscan2; and **d.** DeepVariant. FVC consistently achieves the highest AUC score when applied to both SNV and INDEL variants.

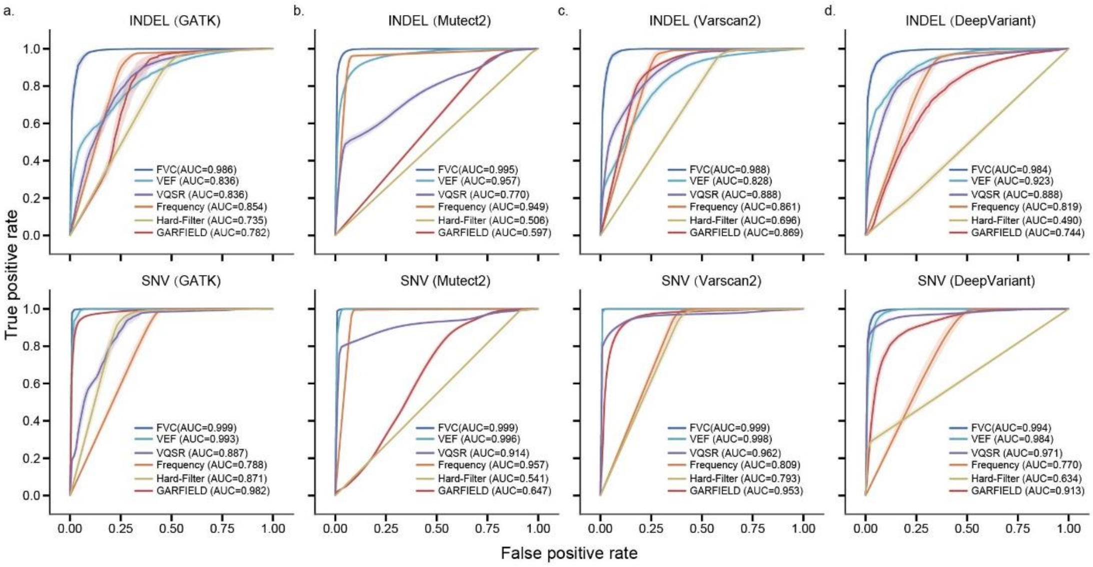

Supplementary Figure 5 The performance of different filtering methods measured by leave-one-chromosome-out cross-validation. The SNV and INDEL variants used as testing data are derived from whole-genome sequencing datasets (HG001, HG003, HG004, HG006, and HG007) at 30x coverage. The performance of different filtering methods is assessed on the SNV and INDEL variants identified by **a.** GATK HaplotypeCaller; **b.** Mutect2; **c.** Varscan2; and **d.** DeepVariant. The assessments are performed by using the leave-one-chromosome-out cross-validation method. The shaded area indicates the 95% confidence intervals (n=22

chromosomes). FVC consistently achieves the highest AUC score when applied to both SNV and INDEL variants.

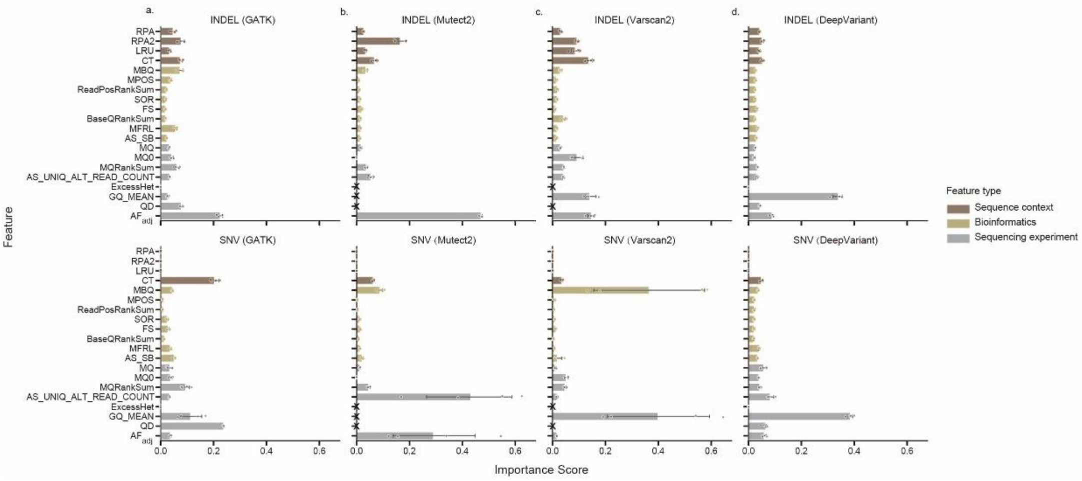

Supplementary Figure 6 Gini importance of features (relative score) when FVC is trained on the variants detected by GATK, Mutect2, Varscan2, and DeepVariant. FVC models are trained by using leave-one-individual-out cross-validation method on the four (HG001, HG003, HG004, HG006) whole-genome sequencing data at 30x coverage. The figure represents the scaled features importance of FVC models when training on the INDEL or SNV variants identified by **a.** GATK HaplotypeCaller, **b.** Mutect2, **c.** Varscan2, and **d.** DeepVariant. The error bar indicates the 95% confidence intervals of scaled importance (n=4 biologically independent samples).

The same feature demonstrates different importance in the SNV model and Indel model and demonstrated different performances in different variant callers. It indicates that researchers should build different filtering models according to the variant types and the variant callers. It is worth noting that the constructed features AF_{adj} and CT have made important contributions to the model construction. The feature AF_{adj} achieved the highest feature importance score in building models for filtering INDEL variants identified by GATK and Mutect2. The feature CT outperforms the other sequence context related features in constructing models for filtering SNV variants identified by all considered variant callers in this study. Maybe they can be used in the further studies.

2. Supplementary Methods

2.1 Datasets

We download the whole genome sequencing data and the gold standard variant call sets from the following ftp.

2.1.1 Sequencing data in BAM format

(HG001)

ftp://ftp-
trace.ncbi.nlm.nih.gov/ReferenceSamples/giab/data/NA12878/NIST_NA12878_HG001_HiSeq_300x/NHGRI_Illumina300X_novoalign_bams/HG001.hs37d5.300x.bam

(HG003 BAM file)
ftp://ftp-
trace.ncbi.nlm.nih.gov/giab/ftp/data/AshkenazimTrio/HG003_NA24149_father/NIST_Illumina_2x250bps/novoalign_bams/HG003.hs37d5.2x250.bam

(HG004 BAM file)
ftp://ftp-
trace.ncbi.nlm.nih.gov/giab/ftp/data/AshkenazimTrio/HG004_NA24143_mother/NIST_Illumina_2x250bps/novoalign_bams/HG004.hs37d5.2x250.bam

(HG006 BAM file)
ftp://ftp-trace.ncbi.nlm.nih.gov/giab/ftp/data/ChineseTrio/HG006_NA24694-huCA017E_father/NA24694_Father_HiSeq100x/NHGRI_Illumina100X_Chinesetrio_novoalign_bams/HG006.hs37d5.100x.bam

(HG007 BAM file)
ftp://ftp-trace.ncbi.nlm.nih.gov/giab/ftp/data/ChineseTrio/HG007_NA24695-hu38168_mother/NA24695_Mother_HiSeq100x/NHGRI_Illumina100X_Chinesetrio_novoalign_bams/HG007.hs37d5.100x.bam

2.1.2 Gold standard variant calls in VCF file

(HG001)
https://ftp-
trace.ncbi.nlm.nih.gov/giab/ftp/release/NA12878_HG001/NISTv3.3.2/GRCh37/HG001_GRCh37_GIAB_highconf_CG-IllFB-IllGATKHC-Ion-10X-SOLID_CHROM1-X_v.3.3.2_highconf_PGandRTGphasetransfer.vcf.gz

(HG003)
https://ftp-
trace.ncbi.nlm.nih.gov/giab/ftp/release/AshkenazimTrio/HG003_NA24149_father/latest/GRCh37/HG003_GRCh37_GIAB_highconf_CG-IllFB-IllGATKHC-Ion-10X_CHROM1-22_v.3.3.2_highconf.vcf.gz

(HG004)
https://ftp-
trace.ncbi.nlm.nih.gov/giab/ftp/release/AshkenazimTrio/HG004_NA24143_mother/latest/GRCh37/HG004_GRCh37_GIAB_highconf_CG-IllFB-IllGATKHC-Ion-10X_CHROM1-22_v.3.3.2_highconf.vcf.gz

(HG006)
https://ftp-
trace.ncbi.nlm.nih.gov/giab/ftp/release/ChineseTrio/HG006_NA24694_father/NISTv3.3.2/GRCh37/HG006_GIAB_GRCh37_highconf_CG-IllFB-IllSNT-10X_CHROM1-22_v.3.3.2_highconf.vcf.gz

(HG007)
https://ftp-
trace.ncbi.nlm.nih.gov/giab/ftp/release/ChineseTrio/HG007_NA24695_mother/NISTv3.3.2/GRCh37/HG007_GIAB_GRCh37_highconf_CG-IllFB-IllSNT-10X_CHROM1-22_v.3.3.2_highconf.vcf.gz

2.2 Commands for variant calling

```
gatk HaplotypeCaller \
    -R human_hg1kv37.fasta \
    -I $input_bam_file \
    -O GATK_out.vcf

gatk Mutect2 \
    -R human_hg1kv37.fasta \
    -I $input_bam_file \
    -O Mutect2_out.vcf

samtools mpileup -B -f human_hg1kv37.fasta $input_bam_file | java -jar VarScan.v2.3.9.jar \
    mpileup2snp \
    --min-var-freq 0.01 \
    --min-coverage 3 \
    --p-value 0.1 \
    --output-vcf 1 \
    > Varscan2_snp.vcf

samtools mpileup -B -f human_hg1kv37.fasta $input_bam_file | java -jar VarScan.v2.3.9.jar \
    mpileup2indel \
    --min-var-freq 0.01 \
    --min-coverage 3 \
    --p-value 0.1 \
    --output-vcf 1 \
    > Varscan2_indel.vcf

singularity exec deepvariant.simg \
    /opt/deepvariant/bin/run_deepvariant \
    --ref=human_hg1kv37.fasta \
    --reads=$input_bam_file \
    --num_shards=$threads \
    --intermediate_results_dir=Temp_folder \
    --output_vcf=deepvariant_out.vcf
```

2.3 Commands for variant filtering

2.3.1 Hard Filter

```
gatk VariantFiltration \
    -V $input_vcf -filter "QD <2.0" --filter-name "QD2" \
    -filter "QUAL <30.0" --filter-name "QUAL30" \
    -filter "SOR > 3.0" --filter-name "SOR3" \
    -filter "FS > 60.0" --filter-name "FS60" \
    -filter "MQ < 40.0" --filter-name "MQ40" \
    -filter "MQRankSum < -12.5" --filter-name "MQRankSum-12.5" \
    -filter "ReadPosRankSum < -8.0" --filter-name "ReadPosRankSum-8" \
    -O hf_filtered.vcf
```

2.3.2 VQSR

```
gatk VariantRecalibrator \
    -V $input_vcf \
    -R human_hg1kv37.fasta \
    --resource hapmap,known=false,training=true,truth=true,prior=15.0:hapmap_3.3.b37.vcf \
    --resource omini,known=false,training=true,truth=false,prior=12.0:1000G_omni2.5.b37.vcf \
    --resource 1000G,known=false,training=true,truth=false,prior=10.0:
        1000G_phase1.snps.high_confidence.b37.vcf \
    --resource dbsnp,known=true,training=false,truth=false,prior=2.0: dbsnp_138.b37.vcf \
    -an MQ -an SOR -an FS -an MQRankSum -an ReadPosRankSum -an QD -an DP \
    --mode SNP \
    --max-gaussians 1 \
    --tranches-file vqsr_snps.tranches \
    -O vqsr_snps_recal.vcf
gatk ApplyVQSR \
    -V $input_vcf \
    -R human_hg1kv37.fasta \
    --truth-sensitivity-filter-level 99.0 \
    --tranches-file vqsr_snps.tranches \
    --recal-file vqsr_snps_recal.vcf \
    --mode SNP \
    -O vqsr_snp_filtered.vcf
gatk VariantRecalibrator \
    -V vqsr_snp_filtered.vcf \
    -R human_hg1kv37.fasta \
    --resource mills,known=true,training=true,truth=true,prior=12.0:
        Mills_and_1000G_gold_standard.indels.b37.vcf \
    --resource dbsnp,known=true,training=false,truth=false,prior=2.0: dbsnp_138.b37.vcf \
    -an MQ -an SOR -an FS -an MQRankSum -an ReadPosRankSum -an QD -an DP \
    --mode INDEL \
    --max-gaussians 1 \
    --tranches-file vqsr_indels.tranches \
    -O vqsr_indels_recal.vcf
gatk ApplyVQSR \
    -V vqsr_snp_filtered.vcf \
    -R human_hg1kv37.fasta \
    --truth-sensitivity-filter-level 99.0 \
    --tranches-file vqsr_indels.tranches \
    --recal-file vqsr_indels_recal.vcf \
    --mode INDEL \
    -O VQSR_ filtered.vcf
```

2.3.3 GARFIELD-NGS

```
perl Predict.pl –input $input_snp_vcf –output GARFIELD_snp_filtered.vcf --platform illumina
perl Predict.pl –input $input_indel_vcf –output GARFIELD_indel_filtered.vcf --platform illumina
```

Then the genetic variant is regarded as false variant if its probability of being correct is less than 0.8369 in SNP or less than 0.630 in INDEL variant.

2.3.4 VEF

```
python hap.py \
    $gold_standard_vcf \
    $input_snp_vcf \
    -f $bed_file \
    -r human_hg1kv37.fasta \
    --set-gt hom
    --no-roc
    -o snp_happy.vcf
python hap.py \
    $gold_standard_vcf \
    $input_indel_vcf \
    -f $bed_file \
    -r human_hg1kv37.fasta \
    --set-gt hom
    --no-roc
    -o indel_happy.vcf
python vef_clf.py \
    --happy snp_happy.vcf \
    --target $Training_snp_vcf \
    --mode SNP \
    --kind vef
python vef_clf.py \
    --happy indel_happy.vcf \
    --target $Training_snp_vcf \
    --mode INDEL \
    --kind vefModel
python vef_apl.py \
    --clf_file vefModel.vef_snp_RF.n_150.clf \
    --subject $input_snp_vcf \
    --mode SNP \
    --suffix vef_filtered_snp
python vef_apl.py \
    --clf_file vefModel.vef_indel_RF.n_150.clf \
    --subject $input_indel_vcf \
    --mode INDEL \
    --suffix vef_filtered_indel
```

2.3.5 Frequency

The genetic variant is regarded as false variant if the variant allelic frequency (VAF) is less than 20% or the number of variant allelic reads is less than 5.

2.4 Characterization of Variant call-sets

Supplementary Table 9. The distribution of variants in each subgroup and variant calling pipeline results.

Software	True positive				False positive			
	Total	low-frequency	hard-to-detect	coding variants	Total	low-frequency	hard-to-detect	coding variants
GATK HaplotypeCaller	3,507,544	0.08% (2,913)	4.38% (153,585)	1.90% (66,541),	7,078	54.22% (3,838)	62.29% (4,409)	2.26% (160)
Mutect2	3,486,692	0.10% (3,506)	4.59% (160,134),	1.90% (66,123)	59,133	72.73% (43,008)	95.13% (56,254)	1.92% (1,133)
Varscan2	3,505,952	0.12% (4,229)	0.32% (11,207),	1.90% (66,456)	51,264	67.36% (34,533)	36.05% (18,480)	1.94% (994)
DeepVariant	3,515,933	0.10% (3,516)	2.37% (83,294)	1.89% (66,627)	1,595	30.80% (491)	55.55% (886)	1.58% (25)

The value in the table is the average number of variants derived from six individuals sequencing data at 30x coverage.

2.4 Features used in FVC

Supplementary Table 10. Intersection between the FVC constructed features and the features in raw input VCF file.

Types	FVC Constructed Features	Features in raw VCF (GATK)	Features in raw VCF (Mutect2)	Features in raw VCF (Varscan2)	Features in raw VCF (DeepVariant)
Sequence context (systematic deviations)	RPA (Number of times tandem repeat unit is repeated for reference allele)	×	√	×	×
	RPA2 (Number of times tandem repeat unit is repeated for alternative allele)	×	√	×	×
	LRU (Length of tandem repeat unit)	×	×	×	×
	CT (Combined variant type around the centric variant, snv+snv, snv+indel, indel+indel, without other variants)	×	×	×	×
	MBQ (Median base quality)	×	√	×	×

Sequencing experiment (random errors)	BaseQRankSum (Z-score from Wilcoxon rank sum test of Alt Vs. Ref base qualities)	√	√	×	×
	MPOS (Median distance from end of read)	×	√	×	×
	ReadPosRankSum (Z-score from Wilcoxon rank sum test of Alt vs. Ref read position bias)	√	√	×	×
	AS_SB (Allele-specific forward/reverse read counts for strand bias tests)	×	√	×	×
	SOR (Symmetric Odds Ratio of 2x2 contingency table to detect strand bias)	×	√	×	×
	FS (Phred-scaled p-value using Fisher's exact test to detect strand bias)	√	√	×	×
	MFRL (Median fragment length of alternative allele)	×	√	×	×
Bioinformatics (analysis bias)	MQ (Mapping quality)	√	√	×	×
	MQ0 (Total number of reads with Zero Mapping Quality)	×	√	×	×
	MQRankSum (Z-score From Wilcoxon rank sum test of Alt vs. Ref read mapping qualities)	√	√	×	×
	AS_UNIQ_ALT_READ_COUNT (Number of reads with unique start and mate end positions for each alt at a variant site)	×	√	×	×
	ExcessHet (Phred-scaled p-value for exact test of excess heterozygosity)	√	× (both not exist)	× (both not exist)	×
	GQ_MEAN (Mean of all genotype quality values)	×	× (both not exist)	×	×
	QD (Variant Quality by Depth)	√	× (both not exist)	× (both not exist)	×
	AF_{adj} (Adjusted variant allele frequency)	×	×	×	×

The sequence context related features were defined to characterize the systematic deviations of the sequencing error. The feature LRU indicated the length of the tandem repeat unit around the genetic variant. The features RPA and RPA2 indicated the number of times a tandem repeat unit was repeated for the reference allele and the alternative allele, respectively. They were constructed or derived from the INFO column in the re-annotated VCF file. The feature CT indicated the combined variant type around 20bp of the target variant (e.g., SNV+SNV,

SNV+Indel, Indel+Indel, and without other variants), it was constructed based on the sequence context around the genetic variant.

The experiment related features were defined to characterize the random errors during the sequencing experiment. The MBQ and BaseQRankSum were were selected to demonstrate the random errors of the sequencing base quality and were immediately derived from the INFO column of the processed VCF file. MPOS, ReadPosRankSum, and MFRL indicated the variant location bias in the short sequencing read and were immediately derived from the INFO column of the processed VCF file. Features SOR, FS, and AS_SB indicated the strand bias during the sequencing experiment and were derived from the INFO column of the VCF file. Among them, AS_SB was newly defined as allele-specific strand bias of forward/reverse read counts in formula (1).

$$\mathrm{AS\_SB} = \frac{\min(\mathrm{AD_{forward}}, \mathrm{AD_{reverse}})}{\max(\mathrm{AD_{forward}}, \mathrm{AD_{reverse}})} \quad (1)$$

Here, $AD_{forward}$ denotes the variant allelic depth in forward orientation; $AD_{reverse}$ denotes the variant allelic depth in reverse orientation.

The bioinformatic analysis related features were defined to characterize the analysis bias from the computational analysis procedure. Features MQ, MQ0, MQRankSum, and AS_UNIQ_ALT_READ_COUNT was derived from INFO column in the processed VCF file to demonstrated the read alignment errors. The GQ_MEAN and QD were selected from INFO column to characterize the statistic bias of the variant confident score. The feature ExcessHet characterized the bias of heterozygosity in a large number of samples. In addition, the variant allele frequency was adjusted by the alternative allelic depth (AD) to minimize the coverage effect when the sequencing depth of the target site is exceptionally high or low (formula (2)).

$$\mathrm{AF_{adj}} = \frac{\mathrm{AD - C}}{\mathrm{AD}} \times \frac{1}{1 + e^{-\mathrm{AF}}} \quad (2)$$

Here, AD denotes the variant allelic depth, AF-the variant allelic frequency. The C denotes the minimum number of variant allelic reads that can be regarded as a reliable variant according to the binomial statistical test. Here we set 2 for default.

Supplementary Table 11. Parameters used in machine learning methods

Method	Parameter	Value	Method	Parameter	Value
Logistic regression	C	1.0	MNP	activation	logistic
	class_weight	None		alpha	0.0001
	dual	False		batch_size	auto
	fit_intercept	True		beta_1	0.9
	intercept_scaling	1		beta_2	0.999
	max_iter	100		early_stopping	False
	multi_class	auto		epsilon	1e-08
	penalty	l2		hidden_layer_sizes	(50, 15)
	solver	lbfgs		learning_rate	constant
	tol	0.0001		max_fun	15000
Random Forest	bootstrap	True		max_iter	200
	ccp_alpha	0.0		momentum	0.9
	criterion	gini		n_iter_no_change	10
	max_features	auto		nesterovs_momentum	True
	min_impurity_decrease	0.0		power_t	0.5
	min_impurity_split	None		random_state	0
	min_samples_leaf	1		shuffle	True
	min_samples_split	2		solver	adam
	oob_score	False		tol	0.0001
	n_estimators	100		validation_fraction	0.1
SVM	C	1.0	XGBoost	objective	binary:logistic
	dual	True		base_score	0.5
	fit_intercept	True		booster	gbtree
	intercept_scaling	1		colsample_bylevel	1
	loss	squared_hinge		colsample_bynode	1
	max_iter	1000		colsample_bytree	1
	multi_class	ovr		gamma	0
	penalty	l2		importance_type	gain
	random_state	0		learning_rate	0.3
	tol	0.0001		max_delta_step	0
LightGBM	boosting_type	gbdt		max_depth	6
	colsample_bytree	1.0		min_child_weight	1
	importance_type	split		n_estimators	200
	learning_rate	0.1		num_parallel_tree	1
	min_child_samples	20		random_state	0
	min_child_weight	0.001		reg_alpha	0
	min_split_gain	0.0		reg_lambda	1
	n_estimators	100		scale_pos_weight	1
	num_leaves	31		subsample	1
	subsample_for_bin	200000		tree_method	exact
