# Supplementary material for: FVC as an adaptive and accurate method for filtering variants from popular NGS analysis pipelines
Source: Commun Biol. 2022 Sep 16;5:975. doi: 10.1038/s42003-022-03397-7 (PMC9481582; doi:10.1038/s42003-022-03397-7)
Supplement: Supplementary file 2 — Description of Additional Supplementary Files [file 42003_2022_3397_MOESM2_ESM.pdf]

Description of Additional Supplementary Files

File name: Supplementary Data 1.
Description: The performance of the candidate methods in each module of FVC.

File name: Supplementary Data 2.
Description: The additional experiment results.

File name: Supplementary Data 3.
Description: The performance of different filtering methods.

File name: Supplementary Data 4.
Description: The subgroup analysis results of different filtering methods.

File name: Supplementary Data 5.
Description: The characterization of the datasets used in this study.
