# Supplementary material for: FVC as an adaptive and accurate method for filtering variants from popular NGS analysis pipelines
Source: Commun Biol. 2022 Sep 16;5:975. doi: 10.1038/s42003-022-03397-7 (PMC9481582; doi:10.1038/s42003-022-03397-7)
Supplement: Supplementary file 8 — Reporting Summary [file 42003_2022_3397_MOESM8_ESM.pdf]

nature research

Corresponding author(s): Hui Lu and Hongyu Zhao

Last updated by author(s): Mar 24, 2022

Reporting Summary

Nature Research wishes to improve the reproducibility of the work that we publish. This form provides structure for consistency and transparency in reporting. For further information on Nature Research policies, see our Editorial Policies and the Editorial Policy Checklist.

Statistics

For all statistical analyses, confirm that the following items are present in the figure legend, table legend, main text, or Methods section.

n/a	Confirmed	
☐	☒	The exact sample size (n) for each experimental group/condition, given as a discrete number and unit of measurement
☐	☒	A statement on whether measurements were taken from distinct samples or whether the same sample was measured repeatedly
☐	☒	The statistical test(s) used AND whether they are one- or two-sided<br>*Only common tests should be described solely by name; describe more complex techniques in the Methods section.*
☒	☐	A description of all covariates tested
☒	☐	A description of any assumptions or corrections, such as tests of normality and adjustment for multiple comparisons
☐	☒	A full description of the statistical parameters including central tendency (e.g. means) or other basic estimates (e.g. regression coefficient) AND variation (e.g. standard deviation) or associated estimates of uncertainty (e.g. confidence intervals)
☐	☒	For null hypothesis testing, the test statistic (e.g. F, t, r) with confidence intervals, effect sizes, degrees of freedom and P value noted<br>*Give P values as exact values whenever suitable.*
☒	☐	For Bayesian analysis, information on the choice of priors and Markov chain Monte Carlo settings
☒	☐	For hierarchical and complex designs, identification of the appropriate level for tests and full reporting of outcomes
☒	☐	Estimates of effect sizes (e.g. Cohen's d, Pearson's r), indicating how they were calculated

Our web collection on statistics for biologists contains articles on many of the points above.

Software and code

Policy information about availability of computer code

Data collection	No software was used for data collection.
Data analysis	The BAM files were converted to paired-end FASTQ format using Samtools(v1.7). Sequencing alignment, marking duplicates, and local realignment were performed using the BWA-MEM, Dedup, and Realigner that were integrated into Sentieon's DNASeq (v201711). Variant calling were performed by using GATK HaplotypeCaller (v4.0.11), Varscan2 (v2.3.9), Mutect2 (integrated in GATK v4.1.9), and DeepVariant(v1.2). True-positive and false-positive variants were labeled by using RTG-vcfeval(v3.1). The variant calls in whole genome sequencing data were filtered by using Frequency, Hard-Filter (integrated in GATK v4.0.11), VQSR (integrated in GATK v4.0.11), GARFIELD-NGS (v1.0), and our developed method FVC (v1.0). All scripts for FVC developing and comparison in this study are publicly available at https://github.com/yyren/FVC

For manuscripts utilizing custom algorithms or software that are central to the research but not yet described in published literature, software must be made available to editors and reviewers. We strongly encourage code deposition in a community repository (e.g. GitHub). See the Nature Research guidelines for submitting code & software for further information.

Data

Policy information about availability of data

All manuscripts must include a data availability statement. This statement should provide the following information, where applicable:

- Accession codes, unique identifiers, or web links for publicly available datasets
- A list of figures that have associated raw data
- A description of any restrictions on data availability

The raw data that support the findings of this study are publicly available in NIST’s GIAB repository (https://github.com/genome-in-a-bottle/giab_data_indexes/tree/

April 2020

master). The processed data that support the findings of this study are committed on the Dryad Digital Repository (https://doi.org/10.5061/dryad.hdr7sqvkm). The source data underlying the graphs are provided within Supplementary Data files 1-5 (excel).

Field-specific reporting

Please select the one below that is the best fit for your research. If you are not sure, read the appropriate sections before making your selection.

☒ Life sciences ☐ Behavioural & social sciences ☐ Ecological, evolutionary & environmental sciences

For a reference copy of the document with all sections, see nature.com/documents/nr-reporting-summary-flat.pdf

Life sciences study design

All studies must disclose on these points even when the disclosure is negative.

Sample size	No sample-size calculation was performed. First of all, all public gold-standard whole genome sequencing data used in this study comprise tens of millions of variant call-sets. Furthermore, hypothesis testing is not necessary for our algorithm, thus the sample size is not a concern for data preprocessing and analysis.
Data exclusions	There are seven (HG001-HG007) individuals in the NIST database, the HG005 individual is the son of HG006 (father) and HG007 (mother), the HG002 is the son of HG003 (father) and HG004 (mother). To further investigate the performance of FVC trained without genetic relative samples, HG002 and HG005 were excluded in our study. Variant calls not located on the cross-validated high-confident regions were also excluded, the exclusions were performed by using RTG-vcfeval software which considered the different representations of same variants.
Replication	The training and testing data were built by applying a leave-one-out cross-sampling method. Sampling were implemented on the four individuals (HG001, HG003, HG004, and HG006) four times. Each time, variant calls from a different individual was left out (regard as testing data), and the remainder of the variant calls (3 out of the 4 individuals) formed the training data. As a result, 4 replicates for each condition were performed to obtain robust estimation for all competing approaches. We then performed the assessment using the leave-one-chromosome-out cross-validation method. Specifically, we used the autosome variants derived from five human samples (HG001, HG003, HG004, HG006, and HG007). Sampling was implemented on the 22 chromosomes 22 times. Each time, a different chromosome was left out, the genetic variants from the left chromosome formed the test data, and the others formed the training data. There is no duplication between the training and testing data. FVC can offer higher accuracy both in minimizing the filtering of true variant calls and maximizing the removal of false variants. Taken together, FVC provided better performance successfully at each replication.
Randomization	Randomization is not relevant to our study. As there is no hypothesis testing between two genetic variant groups when filtering false positive variants in our algorithm. Moreover, the whole genome sequencing variant calls used for testing in the leave-one-out cross-validation measurement were collected from the same individual, and there is no covariate information.
Blinding	Blinding is not relevant to our study. As there is no hypothesis testing between two groups when filtering false positive variant calls in our algorithm.

Reporting for specific materials, systems and methods

We require information from authors about some types of materials, experimental systems and methods used in many studies. Here, indicate whether each material, system or method listed is relevant to your study. If you are not sure if a list item applies to your research, read the appropriate section before selecting a response.

Materials & experimental systems

n/a	Involved in the study
☒	☐ Antibodies
☒	☐ Eukaryotic cell lines
☒	☐ Palaeontology and archaeology
☒	☐ Animals and other organisms
☒	☐ Human research participants
☒	☐ Clinical data
☒	☐ Dual use research of concern

Methods

n/a	Involved in the study
☒	☐ ChIP-seq
☒	☐ Flow cytometry
☒	☐ MRI-based neuroimaging
